# Association between retinal microvascular abnormalities and late-life brain amyloid-β deposition: the ARIC-PET study

**DOI:** 10.1186/s13195-024-01461-4

**Published:** 2024-05-06

**Authors:** Marco Egle, Jennifer A. Deal, Keenan A. Walker, Dean F. Wong, A. Richey Sharrett, Rebecca F. Gottesman

**Affiliations:** 1 National Institute of Neurological Disorders and Stroke Intramural Research Program, National Institutes of Health, Bethesda, MD 20814 USA; 2 Department of Epidemiology, Johns Hopkins Bloomberg School of Public Health, Baltimore, MD 21231 USA; 3 Laboratory of Behavioral Neuroscience, National Institute on Aging, Intramural Research Program, Baltimore, MD 21224 USA; 4 Mallinckrodt Institute of Radiology, Washington University School of Medicine in St. Louis, St. Louis, MO 63110 USA

**Keywords:** Aβ burden, Alzheimer’s disease, Amyloid burden, Biomarkers, Fundus retinal photography, Microvasculature, Positron emission tomography, Retinal markers, Vascular burden

## Abstract

**Background:**

Retinal microvascular signs are accessible measures of early alterations in microvascular dysregulation and have been associated with dementia; it is unclear if they are associated with AD (Alzheimer’s disease) pathogenesis as a potential mechanistic link. This study aimed to test the association of retinal microvascular abnormalities in mid and late life and late life cerebral amyloid.

**Methods:**

Participants from the ARIC‐PET (Atherosclerosis Risk in Communities‐Positron Emission Tomography) study with a valid retinal measure (*N* = 285) were included. The associations of mid- and late-life retinal signs with late-life amyloid-β (Aβ) by florbetapir PET were tested. Two different measures of Aβ burden were included: (1) elevated amyloid (SUVR > 1.2) and (2) continuous amyloid SUVR. The retinal measures’ association with Aβ burden was assessed using logistic and robust linear regression models. A newly created retinal score, incorporating multiple markers of retinal abnormalities, was also evaluated in association with greater Aβ burden.

**Results:**

Retinopathy in midlife (*OR* (95% CI) = 0.36 (0.08, 1.40)) was not significantly associated with elevated amyloid burden. In late life, retinopathy was associated with increased continuous amyloid standardized value uptake ratio (SUVR) (*β* (95%CI) = 0.16 (0.02, 0.32)) but not elevated amyloid burden (*OR* (95%CI) = 2.37 (0.66, 9.88)) when accounting for demographic, genetic and clinical risk factors. A high retinal score in late life, indicating a higher burden of retinal abnormalities, was also significantly associated with increased continuous amyloid SUVR (*β* (95% CI) = 0.16 (0.04, 0.32)) independent of vascular risk factors.

**Conclusions:**

Retinopathy in late life may be an easily obtainable marker to help evaluate the mechanistic vascular pathway between retinal measures and dementia, perhaps acting via AD pathogenesis. Well-powered future studies with a greater number of retinal features and other microvascular signs are needed to test these findings.

**Supplementary Information:**

The online version contains supplementary material available at 10.1186/s13195-024-01461-4.

## Background

Alzheimer’s disease (AD) is a global health problem, and the pathology of β-amyloid (Aβ) is a key feature in the brain triggering its pathogenesis [1]. Recent imaging advances such as positron emission tomography (PET) enables the detection of Aβ in individuals years before the onset of clinical symptoms [2]. It has also been suggested that Aβ burden may be preceded, likely by years, by alterations in cerebrovascular regulation [3]. This concept is supported by previous studies showing that vascular risk factors in midlife and markers of cerebral small vessel disease were significantly associated with Aβ accumulation in AD and in the general population [4–7].

The retinal microvasculature is both anatomically and physiologically similar to the small vessels in the brain, and retinal microvascular markers have been significantly associated with incident clinical stroke, radiological markers of cerebral small vessel disease, and with dementia [8–12]. It is not well understood, however, if retinal microvasculature alters dementia risk via direct impacts on AD pathogenesis. Differences in retinal microvascular network alterations have been observed in association with AD; a recent meta-analysis found significant yet inconsistent pathologic changes in fractal dimension, vessel caliber, and tortuosity in AD patients [13]. Studies employing advanced retinal imaging techniques such as OCT and OCT-A have furthermore shown reduced choroidal thickness in AD patients and an increase in foveal avascular zone in individuals with amyloid-positive pre-clinical AD [14, 15]. A recent study with a retinal photograph-based deep learning algorithm has additionally demonstrated a high accuracy rate in distinguishing between individuals with vs. without a clinical diagnosis of AD and with high vs. low amyloid burden [16]. While the results look promising, these advanced technologies may be less suitable for routine clinical practice in identifying people at risk for the neurodegenerative disease. An alternative approach would be an easily computable retinal score for each patient that comprises well-understood vascular features. It would also allow for a more mechanistic interpretation of the associations with amyloid-beta.

This study evaluates the mid- and late-life independent retinal microvascular contributions to brain amyloid burden in a biracial population-based ancillary study without dementia in late life. It furthermore explores a new approach to summarize four main retinal microvascular signs (i.e., retinopathy, arteriovenous nicking, focal arteriolar narrowing, and generalized retinal arteriolar narrowing) into a single score for each participant. The goal of the study is to explore if retinal measures are linked to AD pathogenesis specifically, defined with the use of amyloid PET imaging.

## Methods

### Study population

The Atherosclerosis Risk in Communities (ARIC) study is a population-based prospective study of 15,792 men and women aged 45–64 years at baseline (1987–1989) from four U.S. communities: Washington County, Maryland; Forsyth County, North Carolina; Jackson, Mississippi; and Minneapolis, Minnesota. Between baseline and the year 2013, participants completed 4 additional in-person visits. These visits were scheduled between midlife, in the years 1990–92 (visit 2), 1993–95 (visit 3) and 1996–98 (visit 4), and late life in the years 2011–13 (visit 5).

**Fig. 1:**
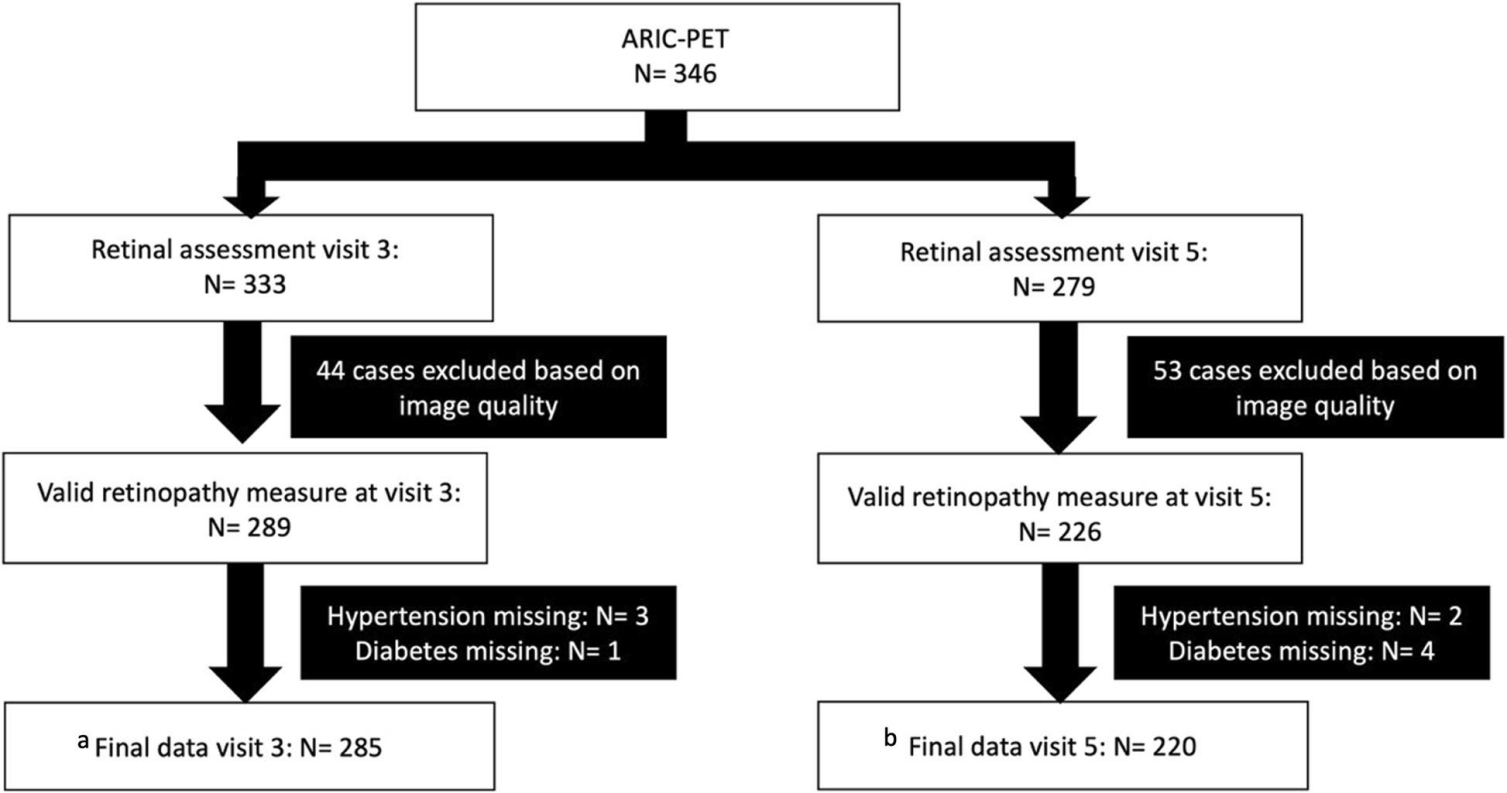
Flow diagram for inclusion in the ARIC-PET retinopathy analytic sample. Cases with a valid retinopathy measure but with missing arteriovenous nicking and generalized arteriolar narrowing measures were imputed. At visit 5, the missing APOE-4 status (*N* = 2) was imputed to retain the maximum number of positive retinopathy cases. ^a^Cases imputed at visit 3: Arteriovenous nicking: *N* = 2; Generalized arteriolar narrowing: *N* = 16. ^b^Cases imputed at visit 5: Arteriovenous nicking: *N* = 2; Generalized arteriolar narrowing: *N* = 16; APOE-4 status: *N* = 2

At visit 5 (late-life; ages 69–89 years), 6538 surviving participants underwent an extensive neuropsychological battery, as part of the ARIC Neurocognitive Study, and individuals were categorized as having normal cognitive function, mild cognitive impairment, or dementia [17]. A subset of the cohort was also selected for research brain MRI. Of those participants included in the MRI imaging sub study, 346 participants from 3 ARIC sites (Jackson, Mississippi; Washington County, Maryland; and Forsyth County, North Carolina) were also recruited for the ancillary ARIC-PET study as previously described [18]. Only individuals without an initial dementia diagnosis, heavy alcohol use, renal dysfunction or prolonged QT-c interval were included. 289 and 226 individuals had sufficient high-quality retinal imaging data to characterize the primary retinal measure of interest, i.e., retinopathy vs. no retinopathy, at visit 3 and visit 5 respectively. Additional participants at visit 3 (*N* = 4) and visit 5 (*N* = 6) were excluded if the diabetes or hypertension measure was missing (Fig. 1). Study procedures were approved by the Institutional Review Board for each field center.

### Neuroimaging

Brain MRIs performed on a 3 T magnet and Florbetapir PET scans were carried out at each ARIC site, within 12 months but ideally within 6 months of each other. Participants without a contraindication of MRI were eligible for the MRI imaging substudy when meeting one of the following criteria: [1] prior brain MRI during an earlier ARIC brain MRI ancillary visit; [2] low scores of cognitive function on an extensive neuropsychological battery or a marked decline on cognition on tests repeated at ARIC visits 2, 4, and 5; or [3] an age-stratified random sample of individuals with normal cognitive function [17]. For the florbetapir PET scans, an isotope was injected through a butterfly needle and images were obtained from 50–70 min for a 20-min uptake scan. PET scans were centrally reviewed and quantified at the PET image analysis center (Johns Hopkins); brain MRI scans, all performed on a 3 T magnet at facilities associated with each field center, were centrally reviewed at the MRI reading center (Mayo Clinic). PET scans were quantified for standardized uptake value ratios (SUVRs) in distinct regions of interest, and co-registered to the MRI images. A global cortical measure of Aβ was acquired by computing the weighted average of the following brain regions: orbitofrontal, prefrontal, and superior frontal cortices, lateral temporal, parietal, and occipital lobes, precuneus, and anterior and posterior cingulate. Based on the sample median, the SUVR was dichotomized at a value of 1.2. Elevated Aβ burden was defined as a global cortical SUVR greater than 1.2.

### Retinal exposures

45° non-stereoscopic color retinal photographs of 2 fields (one centered on the optic nerve and one on the macula) in a single randomly selected eye at visit 3 and in both eyes at visit 5 were acquired by trained technicians using nonmydriatic fundus cameras (Canon CR-45UAF; Canon CR-1 Mark II). At both visits, photographs were graded by certified graders masked to the participants’ characteristics at the Ocular Epidemiology Reading Center (OREC) at the University of Wisconsin-Madison based on written standardized protocol and digital photographic standards. Further details on the retinal image acquisition have been described previously [19, 20]. Four measures of retinal microvascular abnormalities were included: [1] retinopathy; [2] arteriovenous nicking; [3] focal arteriolar narrowing; [4] generalized arteriolar narrowing. The presence of retinopathy was determined employing the modified Airlie House classification, as used in the Early Treatment Diabetic Retinopathy Study (ETDRS) [19]. Retinopathy was classified as present (level of 14–87) vs. absent [10–13]. Retinopathy levels between 14–87 refer to a wide spectrum of vascular abnormalities in the eye which include microaneurysms, hemorrhages, soft and hard exudates, venous beading, neovascularization, and fibrosis. Arteriovenous nicking was seen as “definite” in case at least one venous blood column was tapered on both sides of its crossing underneath an arteriole. Focal arteriolar narrowing was determined as “definite” based on the grading and number of arterioles estimated to be ≥ 50 μm in diameter with a constricted area ≤ 2/3 the width of proximal and distal vessel segments. Generalized arteriolar narrowing was defined as lowest quartile of the central retinal arteriolar equivalent (CRAE). CRAE is quantified on the arteriolar diameters within a pre-specified zone surrounding the optic nerve [21]. The reproducibility of the retinal measures was moderate to good (Cohen’s kappa = 0.45–0.89) [19]. The individual reproducibility estimates were as follows: arteriovenous nicking, kappa = 0.61; focal arteriolar narrowing, kappa = 0.45; retinopathy, kappa = 0.89, CRAE, correlation coefficient = 0.74.

### Other variables

Demographic information such as age, sex, race, and education were self-reported and collected at study baseline (1987–1989). Other variables included in this study were APOE-4 genotype (TaqMan assay; Applied Biosystems, Foster City, CA) as well as status of hypertension (measured systolic blood pressure > 140 mm Hg, diastolic blood pressure > 90 mm Hg or use of antihypertensive medications) and of diabetes (fasting glucose > 125, non-fasting > 200, or self-report of diabetes, diagnosed by a physician, or use of antidiabetic medication) at visit 3 and 5. Cognitive status was classified according to expert-adjudicated cognitive outcomes as described previously in detail [17]. Cognitive functions were categorized into normal, mild cognitive impairment, or dementia by a panel of experts according to standardized criteria [17]. As described previously in detail, a battery of neuropsychological test scores was used to compute a global cognition factor score at visit 5 [22].

### Statistical analysis

The statistical analysis was carried out using R (version 3.6.2). The cohort’s characteristics were summarized by descriptive analysis overall and stratified by retinopathy for visit 3 and visit 5. Using multivariate imputation by chained equations (MICE), missing measures of arteriovenous nicking and generalized retinal arteriolar narrowing at both visits were imputed in those cases where a participant had a valid retinopathy measure, but these other measures were missing. APOE-4 status were imputed in 2 cases at visit 5 to retain the maximum number of positive retinopathy cases for the analysis [23].

A comprehensive retinal scoring system was created that summarizes the progressive manifestations of retinal abnormalities in the vasoconstrictive, sclerotic, and exudative phases into a single measure. Whereas generalized arteriolar narrowing, focal arteriolar narrowing and arteriovenous nicking can be seen as early as in the vasoconstrictive and sclerotic phases, features of retinopathy such as tissue ischemia, microaneurysms, hemorrhages and exudates are observed only in the more advanced exudative phase [24]. The scoring system ranged from 0–3. A score of 3 was given to a participant if retinopathy as assessed by the modified Airlie House classification was evident or all other three retinal signs (i.e., arteriovenous nicking, focal arteriolar narrowing, generalized arteriolar narrowing) were present. Individuals were assigned a score of 2 or 1 when two or one retinal sign(s) other than retinopathy were present, respectively. Participants received a score of 0 if they had none of the 4 retinal microvascular features.

Recognizing that a retinal score version that assumes equivalent weighting of these microvascular retinal abnormalities may not be the optimal measure for assessing greater amyloid burden at visit 5, we also created a weighted score version in a sensitivity analysis in which the four retinal measures received different weights based on their association with amyloid burden. To derive the weights, a ridge logistic regression model was employed with the retinal measures as predictors and elevated amyloid burden (SUVR > 1.2) as the dichotomous outcome. To generalize the weights beyond the sample of the ARIC-PET study, the optimal lambda value of the regularized method was selected that minimized the cross-validation prediction error rate for elevated amyloid burden. The weighted score distribution was normalized and transformed to a whole number score range of 0–3 and these numbers were then assigned to each participant.

In the main analysis, the associations between the retinal measures and elevated Aβ burden were tested using separate logistic regression models. A two-step model building process for adjustment was employed. Covariates in model 1 included age, sex, education, APOE4 status and race. In model 2, the presence of hypertension and diabetes was added as additional confounders. We also ran a statistical power analysis to assess the probability of detecting a significant effect of the retinal measures on amyloid burden in the ARIC-PET study if it truly exists in the population.

To assess whether a significant survival bias exists among individuals with retinopathy, the proportions of retinopathy vs. no retinopathy cases at visit 3 stratified by mortality and dementia conversion by visit 5 were computed in the overall ARIC cohort seen at visit 3. It was furthermore tested whether participants who were later enrolled in the ARIC-PET study at visit 5, which specifically excluded individuals with dementia, had a healthier profile at visit 3 than those participants not being recruited for the ancillary study. This was done by comparing the clinical and demographic characteristics between the 2 groups using the Wilcoxon rank sum and Pearson’s Chi-squared tests.

In a secondary analysis employing a robust linear regression analysis with 2000 bootstrap replicates, we also assessed whether retinal signs and the composite scores at visit 5 were associated with increased amyloid SUVR, defined as a continuous outcome measure. The model was adjusted by the covariates age, sex, education, race, and APOE-4 status in model 1. In model 2, the clinical risk factors hypertension and diabetes were further added to the model. Grouped boxplots were also employed to visualize the distributions of amyloid SUVR by retinal signs and scores. We furthermore assessed the association between continuous CRAE and amyloid SUVR.

## Results

### Characteristics of the study population

285 participants with a median age of 57 years at visit 3 had either a high school or equivalent level of education (44%) or some education more than high school (41%) (Table 1). The proportion of females (57%) included in the study was higher than of males (43%) and a minority had a positive APOE-4 carrier status (at least one allele) (31%). Only a small proportion of participants had diabetes (9.5%) or hypertension (35%) at visit 3. This contrasts with visit 5, where participants with a median of 76 years of age were more likely to have diabetes (34%) or hypertension (69%). Only a few participants had retinopathy at visit 3 (*N* = 11) or at visit 5 *(N* = 14) (Table 1). Participants with retinopathy across all visits showed no or few secondary retinal signs such as arteriovenous nicking, focal arteriolar narrowing or generalized arteriolar narrowing. 67 participants (30%) at visit 5 had mild cognitive impairment (Table 1).

**Table 1:** Demographic and clinical characteristics stratified by retinopathy for visit 3 and visit 5, for ARIC-PET participants

		Retinopathy measure available at visit 3 (*N* = 285)			Retinopathy measure available at visit 5 (*N* = 220)		
		Overall^a^ (*N* = 285)	Retinopathy^a^ (*N* = 11)	No retinopathy^a^ (*N* = 274)	Overall^a^ (*N* = 220)	Retinopathy^a^ (*N* = 14)	No retinopathy^a^ (*N* = 206)
Age (years)		57 (54, 61)	55 (52, 62)	57 (54, 62)	76 (71, 80)	78 (72, 79)	76 (71, 81)
Education							
Less than high school		42 (15%)	4 (36%)	38 (14%)	25 (11%)	0 (0%)	25 (12%)
High school or comparable		126 (44%)	4 (36%)	122 (45%)	102 (46%)	9 (64%)	93 (45%)
At least some college		117 (41%)	3 (27%)	114 (42%)	93 (42%)	5 (36%)	88 (43%)
Sex							
Female		162 (57%)	5 (45%)	157 (57%)	117 (53%)	9 (64%)	108 (52%)
APOE-4							
Positive		89 (31%)	5 (45%)	84 (31%)	66 (30%)	6 (43%)	60 (29%)
Race							
Black		113 (40%)	7 (64%)	106 (39%)	64 (29%)	3 (21%)	61 (30%)
Diabetes							
Present		27 (9.5%)	3 (27%)	24 (8.8%)	74 (34%)	12 (86%)	62 (30%)
Hypertension							
Present		99 (35%)	5 (45%)	94 (34%)	152 (69%)	11 (79%)	141 (68%)
Arteriovenous nicking							
Present		19 (6.7%)	0 (0%)	19 (6.9%)	10 (4.5%)	1 (7.1%)	9 (4.4%)
Focal arteriolar narrowing							
Present		53 (19%)	0 (0%)	53 (19%)	16 (7.3%)	1 (7.1%)	15 (7.3%)
Generalized arteriolar narrowing							
Present		66 (23%)	3 (27%)	63 (23%)	59 (27%)	4 (29%)	55 (27%)
Mild cognitive impairment							
Present		-	-	-	67 (30%)	8 (57%)	59 (29%)
Global cognitive function		-	-	-	-0.69 (-1.39, -0.11)	-0.61 (-1.44, -0.01)	-0.69^c^ (-1.38, -0.13)
Elevated amyloid burden							
Present ^b^		144 (51%)	4 (36%)	140 (51%)	110 (50%)	10 (71%)	100 (49%)
Amyloid SUVR		1.20 (1.12, 1.37)	1.18 (1.09, 1.35)	1.20 (1.12, 1.38)	1.20 (1.11, 1.40)	1.29 (1.19, 1.68)	1.19 (1.11, 1.40)

^a^Median (Q1, Q3); *N*(%); ^b^SUVR for global cortical region > 1.2; ^c^cognition score missing for 1 individual

### Association between retinal signs and scores at visit 3 and elevated Aβ burden

**Fig. 2:**
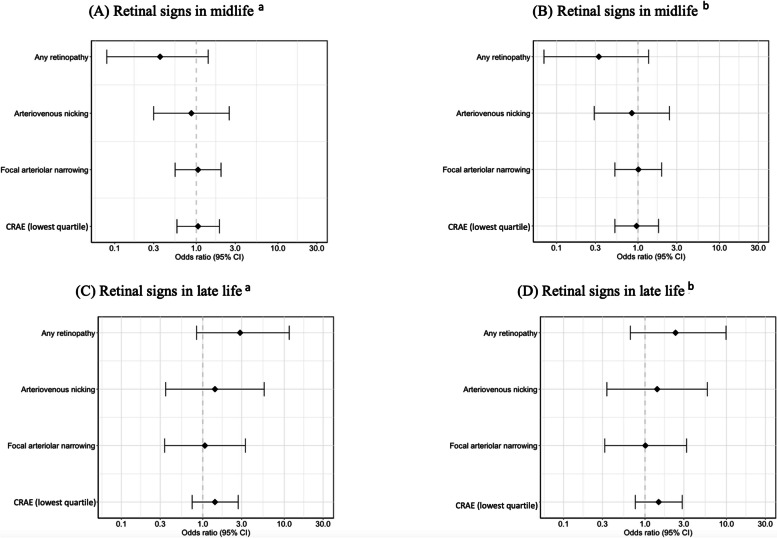
The association between retinal signs in midlife (**A**-**B**), retinal signs in late life (**C**-**D**) and elevated Aβ burden in late life. ^a^Logistic regression model between retinal signs and elevated Aβ burden adjusted by age, sex, education, APOE-4 status, and race. ^b^Logistic regression model between retinal signs and elevated Aβ burden adjusted by age, sex, education, race, APOE-4 status, diabetes, and hypertension

Retinopathy at visit 3 was not significantly associated with elevated Aβ burden at visit 5 in model 1 (*OR* (95% *CI*) = 0.36 (0.08, 1.40)) or in model 2 (*OR* (95% *CI*) = 0.33 (0.07, 1.35)) (Fig. 2). No other retinal signs had a significant association with elevated Aβ burden in either model (Fig. 2).

**Fig. 3:**
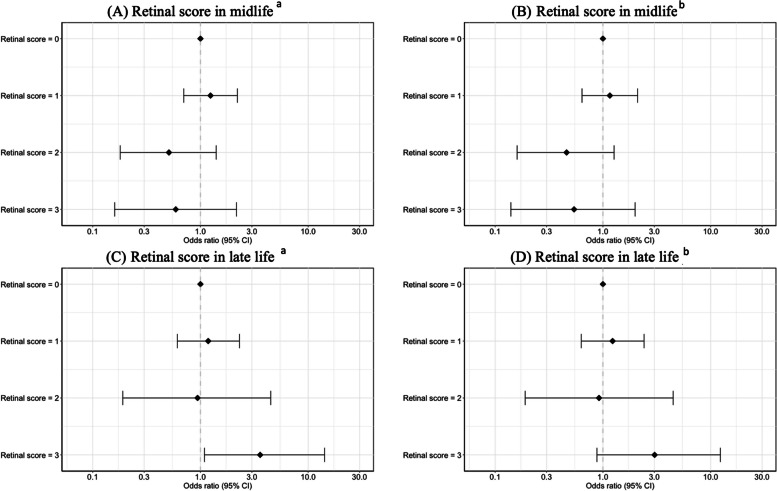
The association between retinal score in midlife (**A**-**B**), retinal score in late life (**C**-**D**) and elevated Aβ burden in late life. ^a^Logistic regression model between retinal signs and elevated Aβ burden adjusted by age, sex, education, APOE-4 status, and race. ^b^Logistic regression model between retinal signs and elevated Aβ burden adjusted by age, sex, education, race, APOE-4 status, diabetes, and hypertension

A total of 164 participants had a composite midlife retinal score of 0, meaning that they showed no retinal signs at visit 3. 87 and 21 individuals had retinal scores of 1 or 2 respectively. In 13 participants, the highest possible retinal score of 3 was given. No significant associations were found when testing whether the retinal scores of 1, 2, or 3 from midlife were related to elevated Aβ burden than a retinal score of 0 (Fig. 3). The full logistic regression models can be found in Supplementary Table 1 and Supplementary Table 2.

### Determining survival bias in the ARIC-PET study

**Fig. 4:**
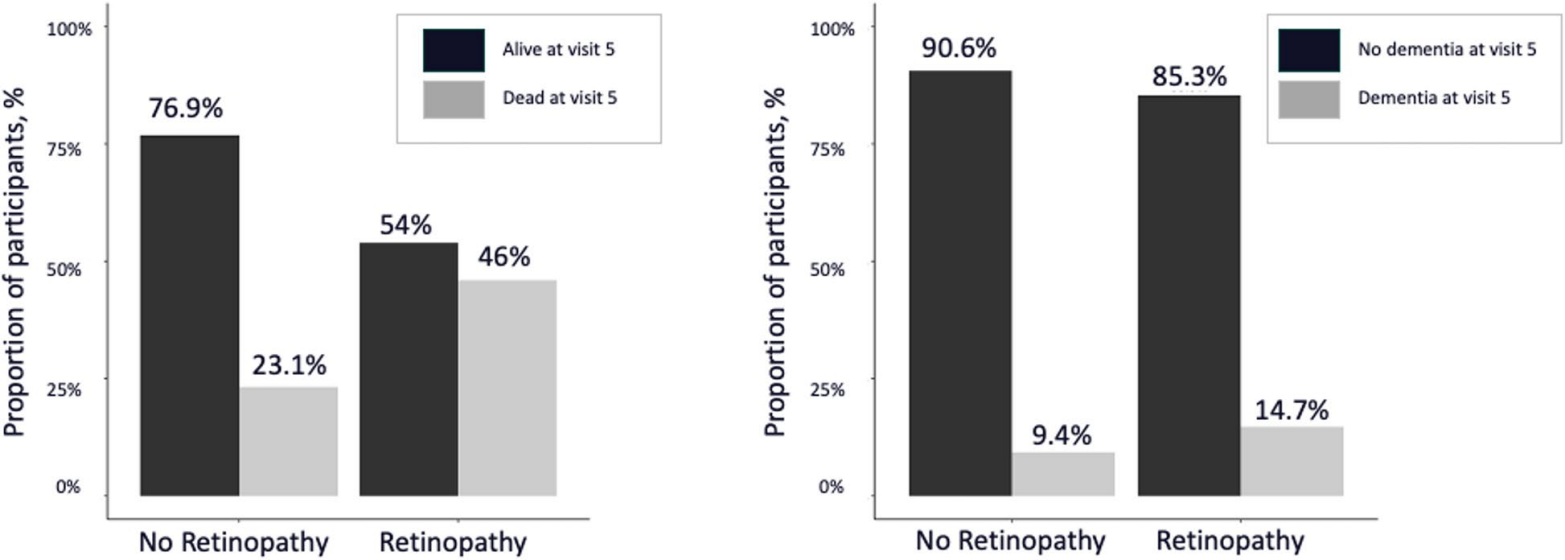
Proportion of participants with retinopathy vs. without retinopathy in midlife being dead or converting to dementia in late life in the overall ARIC cohort (*N* = 11,011)

The low prevalence of retinopathy at visit 3 (*N* = 11) as well as the non-significant association with amyloid burden raised the question of a survival bias. The survival bias was therefore tested in the overall ARIC cohort and participants were included when they had a valid retinopathy measure at visit 3 (*N* = 11,011). The results showed that individuals with retinopathy had a more than twice as high risk of mortality (46% vs. 23.1%) and a significantly higher risk of dementia conversion (14.7% vs. 9.4%) by visit 5 than those participants without the retinal sign (Fig. 4); of note, ARIC-PET participants not only needed to have survived to visit 5 but were excluded if they had prevalent dementia. Differences between participants at visit 3 who were later enrolled in the ARIC-PET and those who were not included in the ancillary study were also found for age and diabetes (Supplementary Table 3). Participants in ARIC-PET were significantly younger and had a lower proportion of diabetes diagnoses at visit 3 than participants who did not enroll in ARIC-PET. A higher proportion of Black participants and a lower proportion of White participants were enrolled in the ARIC-PET study. This difference was primarily driven by the omission of one of the ARIC sites. One field center with predominantly White individuals was not included in the ancillary ARIC-PET study.

### Associations between retinal signs and scores at visit 5 and elevated Aβ burden

The association between retinopathy at visit 5 and elevated Aβ burden was also not significant in model 1 (*OR* (95% *CI*) = 2.87 (0.84, 11.5)) (Fig. 2). The trend remained constant but not significant when accounting for diabetes and hypertension in model 2 (*OR* (95% *CI*) = 2.37 (0.66, 9.88)) (Fig. 2; Supplementary Table 4). There was no significant association with elevated Aβ burden for arteriovenous nicking, focal arteriolar narrowing and generalized arteriolar narrowing (Fig. 2; Supplementary Table 4). The statistical power analysis (δ = 1– β) indicated that retinopathy (*δ* = 0.762), arteriovenous nicking (*δ* = 0.231), focal arteriolar narrowing (*δ* = 0.050), or generalized arteriolar narrowing (*δ* = 0.284) as predictors did not have sufficient power (*δ* < 0.80) to detect a significant effect with elevated amyloid burden in the ARIC-PET study if the effect truly exists in the population (Supplementary Table 5).

139 individuals had a late-life retinal score of 0 indicating that they showed no retinal signs at visit 5 (Fig. 3). Among participants with retinal signs, the majority (*N* = 57; 70.4%) had a score of 1, with 8 having a score of 2 and 16 with a score of 3. When testing whether retinal scores of 1, 2, or 3 in late-life were related to elevated Aβ burden, only the highest retinal score [3] was significantly associated with elevated Aβ burden in model 1 (*OR* (95% *CI*) = 3.58 (1.09, 14.2)) but not in model 2 (*OR* (95% *CI*) = 3.02 (0.88, 12.3)) (Fig. 3), compared to individuals with a retinal score of 0 (no retinal signs). The full logistic regression models can be found in Supplementary Table 6.

In the sensitivity analysis, different weights were assigned to the retinal measures to form a weighted retinal score range (retinopathy, *w* = 1.78; arteriovenous nicking, *w* = -0.02; focal arteriolar narrowing, *w* = 0.51; generalized arteriolar narrowing, *w* = 0.63) (Supplementary Table 7). We did not find a significant association of the weighted retinal scores (1, 2, or 3) in late life with elevated Aβ burden in model 1 or in model 2 (Supplementary Table 8).

### Associations between retinal signs and scores at visit 5 and increased continuous amyloid

The continuous amyloid SUVR stratified by the four microvascular measures can be found in Supplementary Table 9. The amyloid SUVR was higher in those participants with retinopathy and focal arteriolar narrowing, but not with arteriovenous nicking or generalized arteriolar narrowing. We did not observe a linear trend between CRAE and increased amyloid SUVR (Supplementary Fig. 1).

**Fig. 5:**
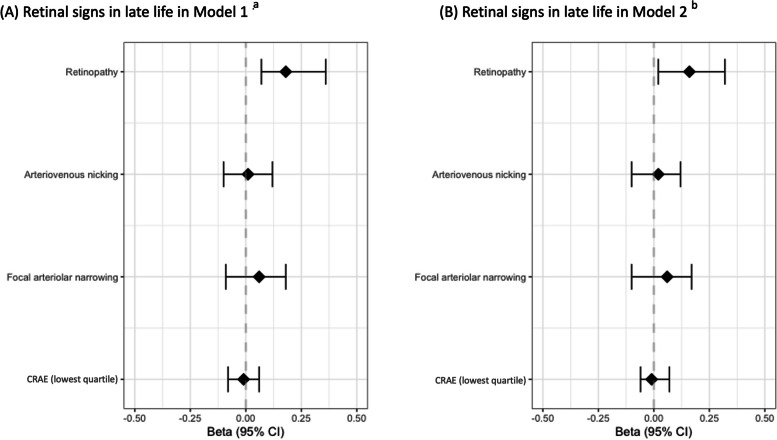
The association between retinal signs in late life and continuous amyloid SUVR in late life in model 1 (**A**) and model 2 (**B**). ^a^Robust linear regression with 2000 bootstrap replicates between retinal signs and SUVR amyloid adjusted by age, sex, education, APOE-4 status, and race. ^b^Robust linear regression with 2000 bootstrap replicates between retinal signs and SUVR amyloid adjusted by age, sex, education, race, APOE-4 status, diabetes, and hypertension

**Fig. 6:**
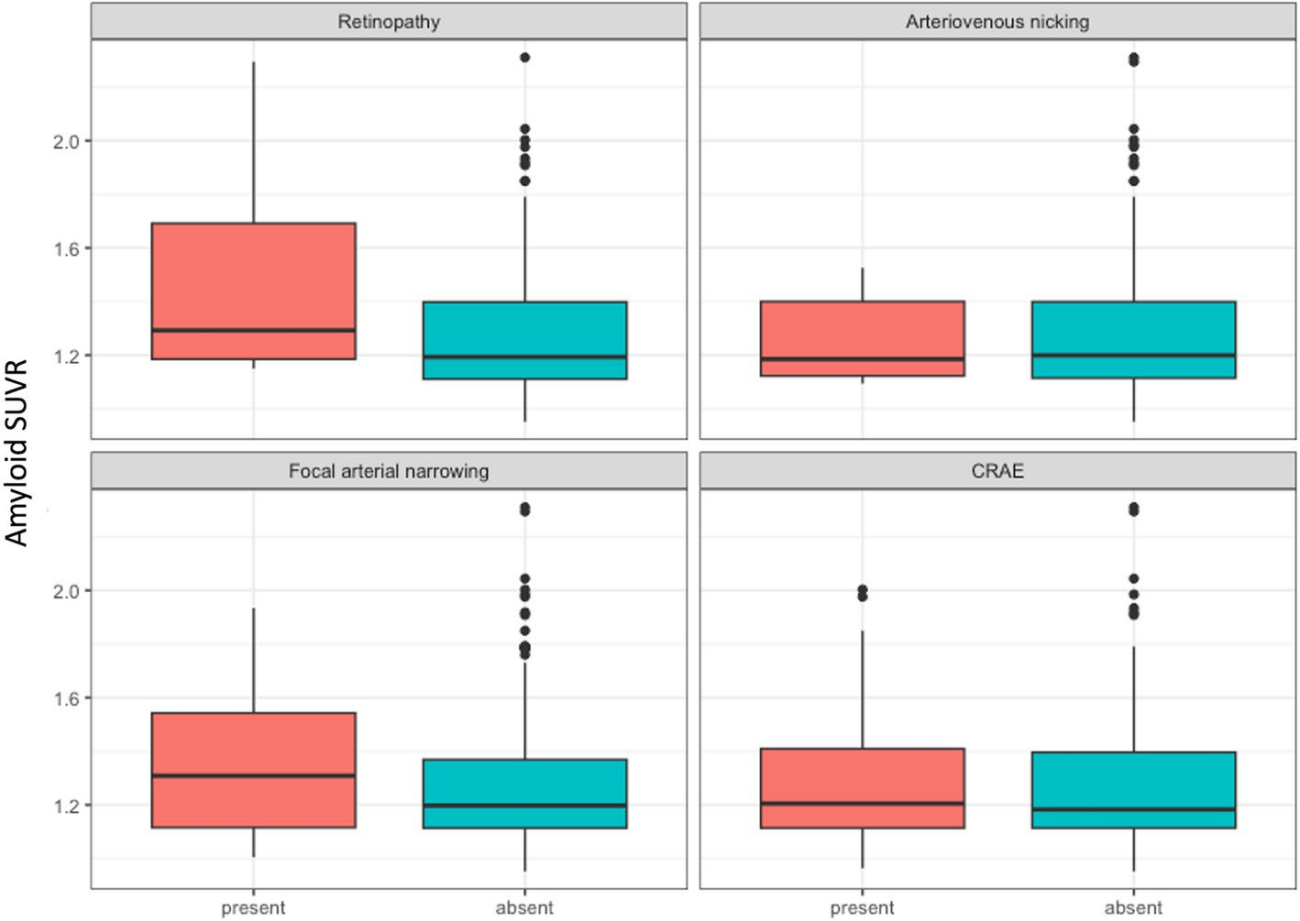
Grouped Boxplot showing the distribution of continuous amyloid SUVR by retinal sign in late life. SUVR- standardized value uptake ratio

Having retinopathy was significantly associated with an increased amyloid SUVR measured continuously when accounting for demographic and genetic covariates in model 1 (*β* (95% *CI*) = 0.18 (0.07, 0.36)) and when further adding the clinical risk factors to the regression in model 2 (*β* (95% *CI*) = 0.16 (0.02, 0.32)) (Fig. 5). Similar associations were not seen for the other 3 retinal signs (Fig. 5; Supplementary Table 10; Fig. 6). Arteriovenous nicking (*β* (95% *CI*) = 0.01 (-0.10, 0.12), focal arteriolar narrowing (*β* (95% *CI*) = 0.06 (-0.09, 0.18)), and generalized arteriolar narrowing (*β* (95% *CI*) = -0.01 (-0.08, 0.06)) were not associated with increased amyloid SUVR. The statistical power analysis (δ = 1– β) indicated that none of the retinal signs were sufficiently powered as predictors (*δ* < 0.80) to detect a significant effect with continuous amyloid SUVR (Supplementary Table 5).

**Fig. 7:**
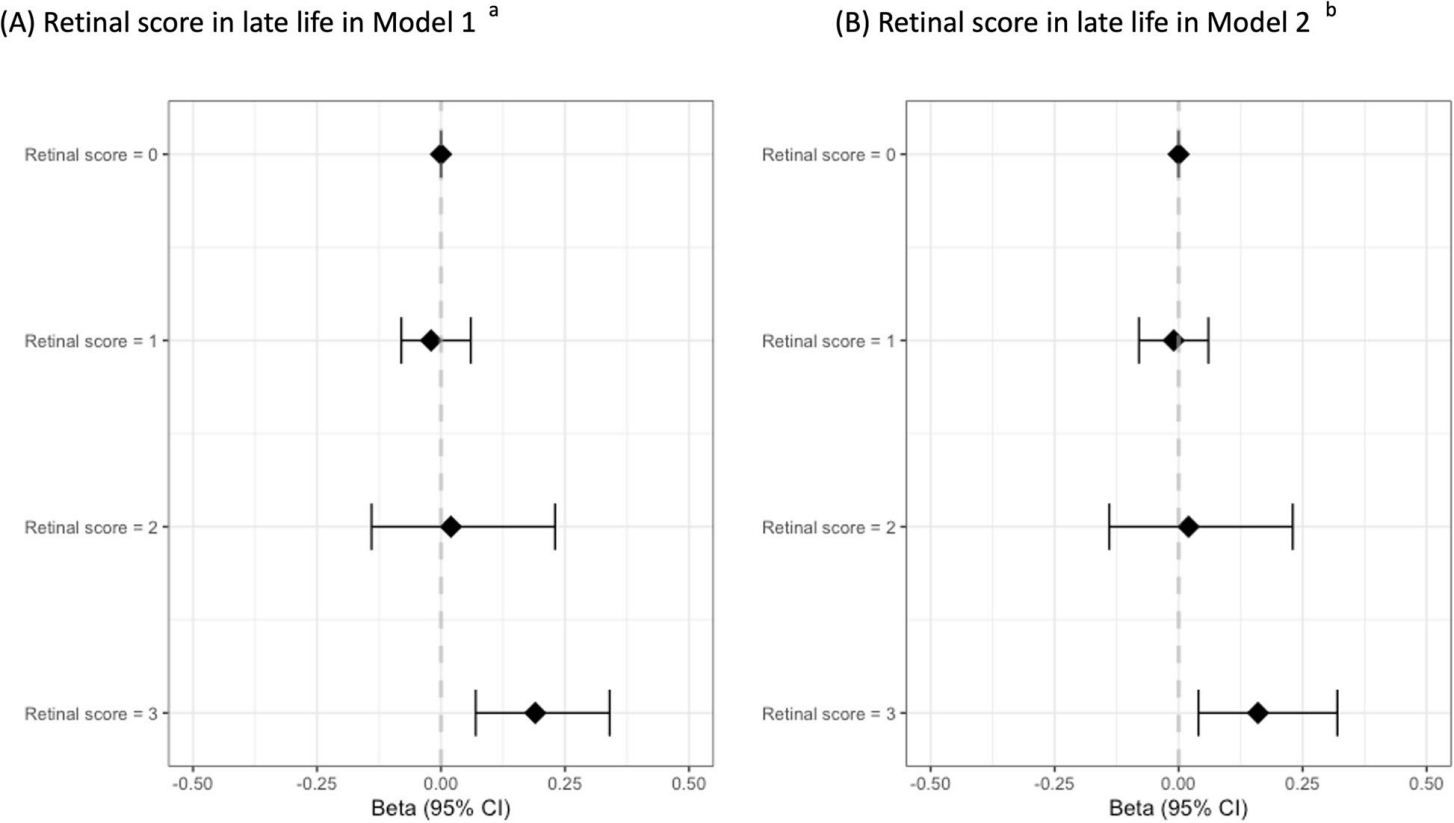
The association between retinal score in late life and continuous amyloid SUVR in late life in model 1 (**A**) and model 2 (**B**). ^a^Robust linear regression with 2000 bootstrap replicates between retinal scores and SUVR amyloid adjusted by age, sex, education, APOE-4 status, and race. ^b^Robust linear regression with 2000 bootstrap replicates between retinal scores and SUVR amyloid adjusted by age, sex, education, race, APOE-4 status, diabetes, and hypertension

A high retinal score of 3 vs. 0 was also significantly associated with an increased continuous amyloid SUVR in model 1 (*β* (95% *CI*) = 0.19 (0.07, 0.34)) and model 2 (*β* (95% *CI*) = 0.16 (0.04, 0.32)) (Fig. 7; Supplementary Table 11; Supplementary Fig. 2). Similar results were found for the weighted retinal score. A high weighted retinal score of 3 vs. 0 was associated with an increased amyloid SUVR in model 1 (*β* (95% *CI*) = 0.18 (0.07, 0.39)) and model 2 (*β* (95% *CI*) = 0.16 (0.03, 0.34)) (Supplementary Table 12).

## Discussion

Contrary to the study’s main hypothesis, retinopathy in midlife was not significantly associated with elevated Aβ burden in late life in this study of individuals without dementia in three U.S. communities. In late life, however, retinopathy was associated with increased amyloid SUVR when analyzed continuously (and independent of existing vascular risk factors hypertension and diabetes), but not associated with an elevated amyloid burden, defined as a SUVR cutoff > 1.2. The results from the exploratory analysis furthermore highlighted a composite retinal score, which incorporates multiple measures of retinal microvascular disease, as a potential risk indicator for elevated and increased Aβ burden in the general population.

Previous evidence has highlighted the role of microvascular retinal lesions relevant to cognitive decline and dementia. Specifically, it has been shown that retinopathy was associated with accelerated rates of 20-year cognitive decline [25]. A more recent study has furthermore demonstrated an association of retinopathy and generalized arteriolar narrowing with all-cause dementia [12]. Multiple mechanisms might link retinal microvascular disease to dementia and cognitive decline, which could include brain microvascular disease, but also might include more direct influences on AD pathogenesis. Retinal microvascular disease is well known to be associated with vascular risk factors such as hypertension and diabetes [24, 26], and it has been suggested that vascular disease burden is a critical factor in the early pathogenesis of AD [3, 7, 27, 28]. Previous studies have demonstrated that vascular risk factors, particularly in midlife, and radiological markers of small cerebral vascular disease (SVD) were significantly associated with higher Aβ burden [4, 5]. Several pathological studies further demonstrated that a large proportion of patients with dementia have mixed vascular and Aβ pathology in the brain [29, 30]. Interestingly, more recent evidence has shown that the vascular and AD pathways may be temporally distinct independently and not synergically contributing to a higher risk of dementia conversion [31–33]. Thus, the consideration of a vascular contribution as evidenced by the retina should include not only its potential impact on AD pathways but also, in future studies, its independent contribution to cognitive outcomes. This study provides some evidence that retinal microvascular disease may be linked to Aβ as a direct (but not only) marker of AD pathogenesis, but overall, we cannot provide conclusive evidence for this association.

This study demonstrates that associations might be best observed when considering a composite measure of overall microvascular disease burden. The idea of summarizing the various vascular lesion types and features occurring at different stages of the disease based on a single score is not new, having been previously applied to cerebral microvascular diseases. A scoring system as we’ve described may be more easily implementable in routine clinical practice than a more complex approach based on deep-learning technology [16]. In cerebral SVD, a high score comprising of the four main MRI radiological features of the disease has been shown to be significantly associated with cognitive impairment and a higher dementia risk in patients with lacunar stroke, with non-symptomatic SVD and in individuals from community-based cohorts [34–36]. It has therefore been suggested that a high SVD score may assist in finding the right target population for a clinical trial [36, 37]. We see similar potential in the retinal field. A high retinal score in late life was the only measure in our study which was significantly associated with both elevated Aβ burden (SUVR > 1.2) as well as increased amyloid SUVR. However, to be more confident about the clinical utility of the retinal scoring system, more studies are needed to independently verify the study’s findings.

The study has several strength and limitations. It is important to highlight the diverse population of Black and White participants from communities in the U.S. as a key strength in this study. One significant limitation, however, is that the number of cases with retinopathy or other retinal signs was low in the ARIC-PET study which likely decreased the statistical power in the analysis. We also recognize that the retinal findings at visit 3 may have suffered from substantial survival bias. Participants were significantly more likely to die or to be diagnosed with dementia in late life when having retinopathy in midlife. As a result, they were less likely to be enrolled in the ancillary ARIC-PET study at visit 5 which is also reflected in the low prevalence of midlife retinopathy.

We also understand that several other confounding factors such as smoking or body mass index may have affected the associations with Aβ burden. Due to the small proportion of retinopathy cases in the ARIC-PET study, we chose not to include these additional covariates in our statistical models. We furthermore acknowledge that using more recently developed software analysis tools such as the Singapore Vessel Analysis (SIVA) would have allowed us to assess a wider spectrum of promising retinal microvascular measures such as fractal dimension of the retinal vessels [38, 39]. Another limitation is that some of the measures had to be imputed in some participants due to some missing retinal variables. Finally, we acknowledge the lack of validation regarding the retinal scoring system used in this study. Future studies may further test its potential value as a measure of overall retinal microvascular health.

In conclusion, this study shows that retinopathy and a high retinal score in late life was significantly associated with increased continuous but not elevated dichotomized Aβ burden above and beyond vascular risk factors in adults without dementia. Retinal microvascular signs in late life may be an easily obtainable marker helping to evaluate the mechanistic vascular pathway between retinal measures and AD pathogenesis. Well-powered future studies with a greater number of participants with retinopathy and other microvascular signs are needed to test these findings and to assess the role of retinal microvascular signs on Aβ burden above and beyond vascular risk factors, and also to evaluate other mechanisms linking retinal microvascular disease with cognitive change and dementia.

### Supplementary Information

**Supplementary Material 1**

## Data Availability

Data that support the findings of this study are available per ARIC (Atherosclerosis Risk in Communities) study policies.

## Abbreviations

AD: Alzheimer’s disease
Aβ: Amyloid-β
ARIC: Atherosclerosis Risk in Communities study
SUVR: Standardized value uptake ratio
SVD: Cerebral small vessel disease
PET: Positron emission tomography
CRAE: Central retinal arteriolar equivalent
MICE: Multivariate imputation by chained equations
OR: Odds ratio
CI: Confidence interval
ETDRS: Early treatment diabetic retinopathy study

## References

[1] Long JM, Holtzman DM (2019). Alzheimer Disease: An Update on Pathobiology and Treatment Strategies. Cell.

[2] Clark CM, Schneider JA, Bedell BJ, Beach TG, Bilker WB, Mintun MA (2011). Use of florbetapir-PET for imaging beta-amyloid pathology. JAMA.

[3] Iturria-Medina Y, Sotero RC, Toussaint PJ, Mateos-Perez JM, Evans AC, Alzheimer's Disease Neuroimaging I (2016). Early role of vascular dysregulation on late-onset Alzheimer's disease based on multifactorial data-driven analysis. Nat Commun.

[4] Gottesman RF, Schneider AL, Zhou Y, Coresh J, Green E, Gupta N (2017). Association Between Midlife Vascular Risk Factors and Estimated Brain Amyloid Deposition. JAMA.

[5] Grimmer T, Faust M, Auer F, Alexopoulos P, Förstl H, Henriksen G (2012). White matter hyperintensities predict amyloid increase in Alzheimer's disease. Neurobiol Aging.

[6] Liu W, Wong A, Law AC, Mok VC (2015). Cerebrovascular disease, amyloid plaques, and dementia. Stroke.

[7] Zlokovic BV (2011). Neurovascular pathways to neurodegeneration in Alzheimer's disease and other disorders. Nat Rev Neurosci.

[8] Patton N, Aslam T, MacGillivray T, Pattie A, Deary IJDB (2005). Retinal vascular image analysis as a potential screening tool for cerebrovascular disease: a rationale based on homology between cerebral and retinal microvasculatures. Anatomical Society of Great Britain and Ireland.

[9] Wong TY, Klein R, Couper DJ, Cooper LS, Shahar E, Hubbard LD (2001). Retinal microvascular abnormalities and incident stroke: the Atherosclerosis Risk in Communities Study. The Lancet.

[10] Hanff TC, Sharrett AR, Mosley TH, Shibata D, Knopman DS, Klein R (2014). Retinal microvascular abnormalities predict progression of brain microvascular disease: an atherosclerosis risk in communities magnetic resonance imaging study. Stroke.

[11] Biffi E, Turple Z, Chung J, Biffi A (2022). Retinal biomarkers of Cerebral Small Vessel Disease: A systematic review. PLoS ONE.

[12] Deal JA, Sharrett AR, Albert M, Bandeen-Roche K, Burgard S, Thomas SD (2019). Retinal signs and risk of incident dementia in the Atherosclerosis Risk in Communities study. Alzheimers Dement.

[13] McGrory S, Cameron JR, Pellegrini E, Warren C, Doubal FN, Deary IJ (2017). The application of retinal fundus camera imaging in dementia: A systematic review. Alzheimers Dement (Amst).

[14] Gharbiya M, Trebbastoni A, Parisi F, Manganiello S, Cruciani F, D'Antonio F (2014). Choroidal thinning as a new finding in Alzheimer's disease: evidence from enhanced depth imaging spectral domain optical coherence tomography. J Alzheimers Dis.

[15] O'Bryhim BE, Apte RS, Kung N, Coble D, Van Stavern GP (2018). Association of Preclinical Alzheimer Disease With Optical Coherence Tomographic Angiography Findings. JAMA Ophthalmol.

[16] Cheung CY, Ran AR, Wang S, Chan VTT, Sham K, Hilal S (2022). A deep learning model for detection of Alzheimer's disease based on retinal photographs: a retrospective, multicentre case-control study. The Lancet Digital Health.

[17] Knopman DS, Gottesman RF, Sharrett AR, Wruck LM, Windham BG, Coker L (2016). Mild Cognitive Impairment and Dementia Prevalence: The Atherosclerosis Risk in Communities Neurocognitive Study (ARIC-NCS). Alzheimers Dement (Amst).

[18] Gottesman RF, Schneider ALC, Zhou Y, Chen X, Green E, Gupta N (2016). The ARIC-PET amyloid imaging study. Neurology.

[19] Hubbard LD, Brothers RJ, King WN, Clegg LX, Klein R, Cooper LS (1999). Methods for evaluation of retinal microvascular abnormalities associated with hypertension/sclerosis in the atherosclerosis risk in communities study. Ophthalmology.

[20] Lee MJ, Deal JA, Ramulu PY, Sharrett AR, Abraham AG (2019). Prevalence of Retinal Signs and Association With Cognitive Status: The ARIC Neurocognitive Study. J Am Geriatr Soc.

[21] Wong TY, Klein R, Sharrett AR, Nieto FJ, Boland LL, Couper DJ (2002). Retinal microvascular abnormalities and cognitive impairment in middle-aged persons: the Atherosclerosis Risk in Communities Study. Stroke.

[22] Gross AL, Power MC, Albert MS, Deal JA, Gottesman RF, Griswold M (2015). Application of Latent Variable Methods to the Study of Cognitive Decline When Tests Change over Time. Epidemiology.

[23] Buuren Sv, Groothuis-Oudshoorn K. MICE: Multivariate Imputation by Chained Equations in R. J Stat Softw. 2011;45(3):1–67.

[24] Cheung CY, Biousse V, Keane PA, Schiffrin EL, Wong TY (2022). Hypertensive eye disease. Nat Rev Dis Primers.

[25] Deal JA, Sharrett AR, Rawlings AM, Gottesman RF, Bandeen-Roche K, Albert M (2018). Retinal signs and 20-year cognitive decline in the Atherosclerosis Risk in Communities Study. Neurology.

[26] Curtis TM, Gardiner TA, Stitt AW (2009). Microvascular lesions of diabetic retinopathy: clues towards understanding pathogenesis?. Eye (Lond).

[27] Sweeney MD, Kisler K, Montagne A, Toga AW, Zlokovic BV (2018). The role of brain vasculature in neurodegenerative disorders. Nat Neurosci.

[28] Kalaria RN, Sepulveda-Falla D (2021). Cerebral Small Vessel Disease in Sporadic and Familial Alzheimer Disease. Am J Pathol.

[29] Schneider JA, Arvanitakis Z, Bang W, Bennett DA (2007). Mixed brain pathologies account for most dementia cases in community-dwelling older persons. Neurology.

[30] Attems J, Jellinger KA (2014). The overlap between vascular disease and Alzheimer's disease - lessons from pathology. BMC Medicine..

[31] Gottesman RF, Wu A, Coresh J, Knopman DS, Jack CR, Rahmim A (2022). Associations of Vascular Risk and Amyloid Burden with Subsequent Dementia. Ann Neurol.

[32] Gold G, Giannakopoulos P, Herrmann FR, Bouras C, Kovari E (2007). Identification of Alzheimer and vascular lesion thresholds for mixed dementia. Brain.

[33] Cogswell PM, Lundt ES, Therneau TM, Mester CT, Wiste HJ, Graff-Radford J (2023). Evidence against a temporal association between cerebrovascular disease and Alzheimer's disease imaging biomarkers. Nat Commun.

[34] Staals J, Booth T, Morris Z, Bastin ME, Gow AJ, Corley J (2015). Total MRI load of cerebral small vessel disease and cognitive ability in older people. Neurobiol Aging.

[35] Huijts M, Duits A, Van Oostenbrugge RJ, Kroon AA, De Leeuw PW, Staals J. Accumulation of MRI markers of cerebral small vessel disease is associated with decreased cognitive function. A study in first-ever lacunar stroke and hypertensive patients. Front Aging Neurosci. 2013;5(72):1–7. 10.3389/fnagi.2013.00072 PMC3818574 24223555

[36] Olama A Amin Al, Wason JMS, Tuladhar AM, van Leijsen EMC, Koini M, Hofer E (2020). imple MRI score aids prediction of dementia in cerebral small vessel disease. Neurology.

[37] Markus HS, van Der Flier WM, Smith EE, Bath P, Biessels GJ, Briceno E (2022). Framework for Clinical Trials in Cerebral Small Vessel Disease (FINESSE): A Review. JAMA Neurol.

[38] McGrory S, Ballerini L, Doubal FN, Staals J, Allerhand M, Valdes-Hernandez MDC (2019). Retinal microvasculature and cerebral small vessel disease in the Lothian Birth Cohort 1936 and Mild Stroke Study. Sci Rep.

[39] Liew G, Wang JJ, Cheung N, Zhang YP, Hsu W, Lee ML (2008). The retinal vasculature as a fractal: methodology, reliability, and relationship to blood pressure. Ophthalmology.

