# Supplementary material for: Association between retinal microvascular abnormalities and late-life brain amyloid-β deposition: the ARIC-PET study
Source: Alzheimers Res Ther. 2024 May 6;16:100. doi: 10.1186/s13195-024-01461-4 (PMC11071225; doi:10.1186/s13195-024-01461-4)
Supplement: Supplementary file 1 — Supplementary Material 1 [file 13195_2024_1461_MOESM1_ESM.docx]

**SUPPLEMENTARY MATERIALS**

**Supplementary Table 1 Associations, in separate models, between retinal signs at visit 3 and elevated amyloid burden at visit 5**. Model 1 was adjusted by the covariates age, sex, education, race and APOE-4 status. In Model 2 diabetes and hypertension were added as additional confounders at visit 3.

|  | Elevated amyloid burden, SUVR > 1.2 | | | | | | | |
| --- | --- | --- | --- | --- | --- | --- | --- | --- |
|  | Model 1 | | | | Model 2 | | | |
|  | Odds ratio  (95% CI) | Odds ratio  (95% CI) | Odds ratio  (95% CI) | Odds ratio  (95% CI) | Odds ratio  (95% CI) | Odds ratio  (95% CI) | Odds ratio  (95% CI) | Odds ratio  (95% CI) |
| Age | **1.10**  **(1.05, 1.16)** | **1.10**  **(1.05, 1.16)** | **1.10**  **(1.05, 1.16)** | **1.10**  **(1.05, 1.16)** | **1.10**  **(1.04, 1.16)** | **1.04**  **(1.04, 1.16)** | **1.04**  **(1.04, 1.16)** | **1.04**  **(1.04, 1.16)** |
| Education |  |  |  |  |  |  |  |  |
| Less than high school | - | - | - | - | - | - | - | - |
| High school or comparable | 0.56  (0.25, 1.20) | 0.60  (0.27, 1.27) | 0.59  (0.27, 1.26) | 0.59  (0.27, 1.26) | 0.59  (0.27, 1.29) | 0.63  (0.29, 1.37) | 0.62  (0.28, 1.35) | 0.62  (0.28, 1.35) |
| At least some college | 0.73  (0.32, 1.59) | 0.79  (0.35, 1.72) | 0.78  (0.35, 1.68) | 0.78  (0.35, 1.69) | 0.74  (0.33, 1.64) | 0.81  (0.36, 1.78) | 0.79  (0.36, 1.73) | 0.79  (0.36, 1.73) |
| Sex |  |  |  |  |  |  |  |  |
| Female | - | - | - | - | - | - | - | - |
| Male | 0.67  (0.39, 1.13) | 0.65  (0.39, 1.10) | 0.65  (0.38, 1.09) | 0.65  (0.38, 1.09) | 0.68  (0.40, 1.16) | 0.67  (0.39, 1.14) | 0.67  (0.39, 1.13) | 0.67  (0.39, 1.13) |
| APOE-4 |  |  |  |  |  |  |  |  |
| Carrier | **2.86**  **(1.64, 5.08)** | **2.80**  **(1.61, 4.95)** | **2.80**  **(1.61, 4.95)** | **2.79**  **(1.60, 4.93)** | **2.98**  **(1.70, 5.33)** | **2.91**  **(1.67, 5.19)** | **2.90**  **(1.66, 5.17)** | **2.91**  **(1.66, 5.18)** |
| Race |  |  |  |  |  |  |  |  |
| Black | **2.25**  **(1.33, 3.85)** | **2.18**  **(1.29, 3.72)** | **2.15**  **(1.28, 3.66)** | **2.16**  **(1.28, 3.68)** | **2.09**  **(1.22, 3.62)** | **2.03**  **(1.19, 3.51)** | **2.01**  **(1.18, 3.46)** | **2.01**  **(1.17, 3.46)** |
| Diabetes |  |  |  |  |  |  |  |  |
| Present | - | - | - | - | 1.15  (0.47, 2.87) | 1.11  (0.45, 2.77) | 1.10  (0.45, 2.73) | 1.11  (0.45, 2.75) |
| Hypertension |  |  |  |  |  |  |  |  |
| Present | - | - | - | - | 1.44  (0.83, 2.51) | 1.42  (0.82, 2.48) | 1.42  (0.81, 2.47) | 1.43  (0.81, 2.52) |
| Any retinopathy |  |  |  |  |  |  |  |  |
| Present | 0.36  (0.08, 1.40) |  |  |  | 0.33  (0.07, 1.35) |  |  |  |
| Arteriovenous nicking |  |  |  |  |  |  |  |  |
| Present |  | 0.87  (0.30, 2.53) |  |  |  | 0.84  (0.29, 2.43) |  |  |
| Focal arterial narrowing |  |  |  |  |  |  |  |  |
| Present |  |  | 1.05  (0.55, 2.01) |  |  |  | 1.01  (0.52, 1.95) |  |
| Generalized arteriolar narrowing |  |  |  |  |  |  |  |  |
| Present |  |  |  | 1.10  (0.60, 2.04) |  |  |  | 1.00  (0.53, 1.89) |

**Statistically significant associations p <0.05 marked in bold*

**Supplementary Table 2. Association between retinal scores at visit 3 and elevated amyloid burden at visit 5**. Model 1 was adjusted by age, sex, education, race, APOE-4. The association in model 2 was further adjusted by diabetes and hypertension.

|  | Elevated amyloid burden, SUVR > 1.2 | |
| --- | --- | --- |
|  | Model 1 | Model 2 |
|  | Odds ratio  (95% CI) | Odds ratio  (95% CI) |
| Age | **1.10**  **(1.05, 1.16)** | **1.10**  **(1.04, 1.16)** |
| Education |  |  |
| Less than high school | - | - |
| High school or comparable | 0.56  (0.26, 1.21) | 0.59  (0.27, 1.29) |
| At least some college | 0.74  (0.33, 1.62) | 0.76  (0.34, 1.66) |
| Sex |  |  |
| Female | - | - |
| Male | 0.64  (0.37, 1.08) | 0.66  (0.38, 1.13) |
| APOE-4 |  |  |
| Carrier | **2.85**  **(1.63, 5.07)** | **3.00**  **(1.71, 5.37)** |
| Race |  |  |
| Black | **2.29**  **(1.35, 3.94)** | **2.12**  **(1.23, 3.68)** |
| Diabetes |  |  |
| Present | **-** | 0.95  (0.38, 2.40) |
| Hypertension |  |  |
| Present | **-** | 0.67  (0.37, 1.17) |
| Retinal scores |  |  |
| Retinal score = 0 | 1 (Ref) | - |
| Retinal score = 1 | 1.24  (0.70, 2.20) | 1.16  (0.64, 2.10) |
| Retinal score= 2 | 0.51  (0.18, 1.40) | 0.46  (0.16, 1.27) |
| Retinal score= 3 | 0.59  (0.16, 2.16) | 0.54  (0.14, 1.99) |

**Statistically significant associations p <0.05 marked in bold*

**Supplementary Table 3**. **Differences in health profiles at visit 3 between participants with a valid retinopathy measure at visit 3, who were recruited into ARIC-PET study at visit 5, and individuals with a valid retinopathy measure in the overall ARIC study at visit 3 who were not later recruited for the ancillary PET study.** Three sites were part of the overall ARIC study as well as the ARIC-PET study: Jackson, Mississippi; Washington County, Maryland; and Forsyth County, North Carolina. Individuals at the Minneapolis, Minnesota ARIC-site did not undergo PET imaging.

| Characteristics at visit 3 | Overall*  (*N*= 11011) | Without PET^a^  (*N*= 10726) | With PET^a^  (*N*= 285) | P-value^b^ |
| --- | --- | --- | --- | --- |
| Age | 59 (55, 64) | 59 (55, 64) | 57 (54, 61) | **< 0.001** |
| Sex |  |  |  |  |
| Female | 6160 (56%) | 5998 (56%) | 162 (57%) | 0.8 |
| Male | 4851 (44%) | 4728 (44%) | 123 (43%) |  |
| Education |  |  |  | 0.2 |
| Less than high school | 2107 (19%) | 2065 (19%) | 42 (15%) |  |
| High school | 4641 (42%) | 4515 (42%) | 126 (44%) |  |
| At least some college | 4245 (39%) | 4128 (39%) | 117 (41%) |  |
| Unknown | 18 | 18 | 0 |  |
| APOE-4 |  |  |  | 0.7 |
| Carrier | 3191 (30%) | 3102 (30%) | 89 (31%) |  |
| Unknown | 447 | 447 | 0 |  |
| Race |  |  |  | **< 0.001** |
| Black | 2380 (22%) | 2267 (21%) | 113 (40%) |  |
| Non-Black | 8631 (78%) | 8459 (79%) | 172 (60%) |  |
| Diabetes |  |  |  | **0.01** |
| Present | 1607 (15%) | 1580 (15%) | 27 (9.5%) |  |
| Unknown | 56 | 56 | 0 |  |
| Hypertension |  |  |  | 0.07 |
| Present | 4384 (40%) | 4285 (40%) | 99 (35%) |  |
| Unknown | 54 | 54 | 0 |  |
| Any retinopathy |  |  |  | 0.13 |
| Retinopathy | 654 (5.9%) | 643 (6.0%) | 11 (3.9%) |  |

^a^Median (Q1, Q3); n (%)

^b^Wilcoxon rank sum test; Pearson's Chi-squared test; Fisher's exact test

**Supplementary Table 4. Associations, in separate models, between retinal signs at visit 5 and elevated amyloid burden at visit 5.** Model 1 was adjusted by the covariates age, sex, education, race and APOE-4 status. In Model 2 the clinical conditions diabetes and hypertension at visit 5 were also added.

|  | Elevated amyloid burden, SUVR > 1.2 | | | | | | | |
| --- | --- | --- | --- | --- | --- | --- | --- | --- |
|  | Model 1 | | | | Model 2 | | | |
|  | Odds ratio  (95% CI) | Odds ratio  (95% CI) | Odds ratio  (95% CI) | Odds ratio  (95% CI) | Odds ratio  (95% CI) | Odds ratio  (95% CI) | Odds ratio  (95% CI) | Odds ratio  (95% CI) |
| Age | 1.03  (0.97, 1.08) | 1.03  (0.97, 1.08) | 1.02  (0.97, 1.08) | 1.02  (0.97, 1.08) | **1.02**  **(0.97, 1.08)** | **1.02**  **(0.97, 1.08)** | **1.02**  **(0.97, 1.08)** | **1.02**  **(0.96, 1.07)** |
| Education |  |  |  |  |  |  |  |  |
| Less than high school | - | - | - | - | - | - | - | - |
| High school or comparable | 0.60  (0.23, 1.53) | 0.65  (0.25, 1.65) | 0.65  (0.25, 1.66) | 0.60  (0.22, 1.55) | 0.64  (0.24, 1.65) | 0.69  (0.27, 1.77) | 0.69  (0.27, 1.77) | 0.63  (0.24, 1.65) |
| At least some college | 0.81  (0.31, 2.12) | 0.87  (0.33, 2.25) | 0.86  (0.33, 2.23) | 0.81  (0.30, 2.11) | 0.86  (0.33, 2.25) | 0.91  (0.35, 2.37) | 0.91  (0.35, 2.37) | 0.85  (0.32, 2.24) |
| Sex |  |  |  |  |  |  |  |  |
| Female | - | - | - | - | - | - | - | - |
| Male | 0.65  (0.36, 1.15) | 0.63  (0.35, 1.12) | 0.64  (0.36, 1.13) | 0.63  (0.35, 1.12) | 0.64  (0.36, 1.15) | 0.64  (0.35, 1.13) | 0.64  (0.35, 1.13) | 0.63  (0.35, 1.12) |
| APOE-4 |  |  |  |  |  |  |  |  |
| Carrier | **3.23**  **(1.72, 6.23)** | **3.32**  **(1.78, 6.38)** | **3.29**  **(1.77, 6.33)** | **3.30**  **(1.77, 6.35)** | **3.34**  **(1.77, 6.50)** | **3.42**  **(1.82, 6.62)** | **3.42**  **(1.82, 6.62)** | **3.44**  **(1.82, 6.68)** |
| Race |  |  |  |  |  |  |  |  |
| Black | **2.15**  **(1.15, 4.10)** | **2.09**  **(1.12, 3.98)** | **2.07**  **(1.10, 3.95)** | **2.06**  **(1.10, 3.92)** | **2.05**  **(1.07, 4.00)** | **2.00**  **(1.04, 3.90)** | **2.00**  **(1.04, 3.90)** | **2.00**  **(1.04, 3.89)** |
| Diabetes |  |  |  |  |  |  |  |  |
| Present | - | - | - | - | 1.35  (0.71, 2.57) | 1.52  (0.82, 2.83) | 1.52  (0.82, 2.83) | 1.57  (0.85, 2.95) |
| Hypertension |  |  |  |  |  |  |  |  |
| Present | - | - | - | - | 1.22  (0.64, 2.35) | 1.22  (0.63, 2.34) | 1.22  (0.63, 2.34) | 1.20  (0.62, 2.31) |
| Any retinopathy |  |  |  |  |  |  |  |  |
| Present | 2.87  (0.84, 11.5) |  |  |  | 2.37  (0.66, 9.88) |  |  |  |
| Arteriovenous nicking |  |  |  |  |  |  |  |  |
| Present |  | 1.41  (0.35, 5.67) |  |  |  | 1.41  (0.34, 5.82) |  |  |
| Focal arterial narrowing |  |  |  |  |  |  |  |  |
| Present |  |  | 1.06  (0.34, 3.33) |  |  |  | 1.01  (0.32, 3.24) |  |
| Generalized arteriolar narrowing |  |  |  |  |  |  |  |  |
| Present |  |  |  | 1.41  (0.74, 2.72) |  |  |  | 1.47  (0.76, 2.86) |

**Statistically significant associations p <0.05 marked in bold*

**Supplementary Table 5. Statistical power analysis given the expected effect sizes**

**Elevated amyloid burden**

| **Retinal measures at visit 5** | **Statistical power** |
| --- | --- |
| Retinopathy | 0.762 |
| Arteriovenous nicking | 0.231 |
| Focal arteriolar narrowing | 0.050 |
| Generalized arteriolar narrowing | 0.284 |

**Continuous amyloid SUVR**

| **Retinal measures at visit 5** | **Statistical power** |
| --- | --- |
| Retinopathy | 0.767 |
| Arteriovenous nicking | 0.061 |
| Focal arteriolar narrowing | 0.186 |
| Generalized arteriolar narrowing | 0.050 |

**Supplementary Table 6.** **Association between retinal scores at visit 5 and elevated amyloid burden at visit 5**. Model 1 was adjusted by age, sex, education, race, APOE-4. The association in model 2 was further adjusted by diabetes and hypertension.

|  | Elevated amyloid burden, SUVR > 1.2 | |
| --- | --- | --- |
|  | Model 1 | Model 2 |
|  | Odds ratio  (95% CI) | Odds ratio  (95% CI) |
| Age | 1.03  (0.97, 1.08) | 1.02  (0.97, 1.08) |
| Education |  |  |
| Less than high school | - | - |
| High school or comparable | 0.57  (0.22, 1.48) | 0.61  (0.23, 1.59) |
| At least some college | 0.80  (0.30, 2.09) | 0.84  (0.32, 2.21) |
| Sex |  |  |
| Female | - | - |
| Male | 0.64  (0.36, 1.15) | 0.64  (0.35, 1.14) |
| APOE-4 |  |  |
| Carrier | **3.20**  **(1.70, 6.20)** | **3.32**  **(1.75, 6.48)** |
| Race |  |  |
| Black | **2.17**  **(1.15, 4.19)** | **2.10**  **(1.08, 4.14)** |
| Diabetes |  |  |
| Present | **-** | 0.74  (0.39, 1.43) |
| Hypertension |  |  |
| Present | **-** | 0.83  (0.43, 1.60) |
| Retinal scores |  |  |
| Retinal score = 1 | 1.18  (0.61, 2.30) | 1.23  (0.63, 2.41) |
| Retinal score= 2 | 0.94  (0.19, 4.49) | 0.92  (0.19, 4.49) |
| Retinal score= 3 | **3.58**  **(1.09, 14.2)** | 3.02  (0.88, 12.3) |

**Statistically significant associations p <0.05 marked in bold*

**Supplementary Table 7. Weights assigned to the retinal measures to create the weighted retinal score**

| **Retinal measures** | **Weights**  **(normalized to a score range of (0-3)** |
| --- | --- |
| Retinopathy | 1.78 |
| Arteriovenous nicking | -0.02 |
| Focal arteriolar narrowing | 0.51 |
| Generalized arteriolar narrowing | 0.63 |

**Supplementary Table 8.** **Association between weighted retinal scores at visit 5 and elevated amyloid burden at visit 5**. Model 1 was adjusted by age, sex, education, race, APOE-4. The association in model 2 was further adjusted by diabetes and hypertension.

|  | Elevated amyloid burden, SUVR > 1.2 | |
| --- | --- | --- |
|  | Model 1 | Model 2 |
|  | Odds ratio  (95% CI) | Odds ratio  (95% CI) |
| Age | 1.02  (0.97, 1.08) | 1.02  (0.97, 1.08) |
| Education |  |  |
| less than high school | — | — |
| high school or comparable | 0.57  (0.22, 1.49) | 0.61  (0.23, 1.60) |
| at least some college | 0.79  (0.29, 2.07) | 0.83  (0.31, 2.19) |
| sex |  |  |
| Female | — | — |
| Male | 0.65  (0.36, 1.16) | 0.65  (0.36, 1.15) |
| APOE-4 |  |  |
| carrier | 3.23  (1.72, 6.25) | 3.35  (1.77, 6.54) |
| Race |  |  |
| Black | 2.17  (1.15, 4.18) | 2.08  (1.08, 4.10) |
| Diabetes |  |  |
| present |  | 1.39  (0.73, 2.68) |
| Hypertension |  |  |
| present |  | 1.20  (0.62, 2.32) |
| Weighted retinal score |  |  |
| Retinal score = 0 | — | — |
| Retinal score = 1 | 1.33  (0.68, 2.64) | 1.38  (0.70, 2.74) |
| Retinal score = 2 | 1.01  (0.21, 4.75) | 0.99  (0.20, 4.79) |
| Retinal score = 3 | 3.10  (0.90, 12.6) | 2.54  (0.70, 10.7) |

**Supplementary Table 9.** Continuous amyloid SUVR stratified by presence of the microvascular measures at visit 5

|  | Frequency, N | Continuous floretapir global cortical SUVR  Median (Q1, Q3) |
| --- | --- | --- |
| Retinopathy |  |  |
| present | 14 | 1.29 (1.19, 1.68) |
| absent | 206 | 1.19 (1.11, 1.40) |
| Arteriovenous nicking |  |  |
| present | 9 | 1.17 (1.11, 1.24) |
| absent | 211 | 1.20 (1.12, 1.40) |
| Focal arteriolar narrowing |  |  |
| present | 16 | 1.31 (1.12, 1.54) |
| absent | 204 | 1.20 (1.11, 1.37) |
| Generalized arteriolar narrowing |  |  |
| present | 54 | 1.20 (1.11, 1.40) |
| absent | 166 | 1.19 (1.11, 1.40) |

**Supplementary Figure 1.** Scatterplot showing no linear trend between continuous CRAE and continuous amyloid SUVR


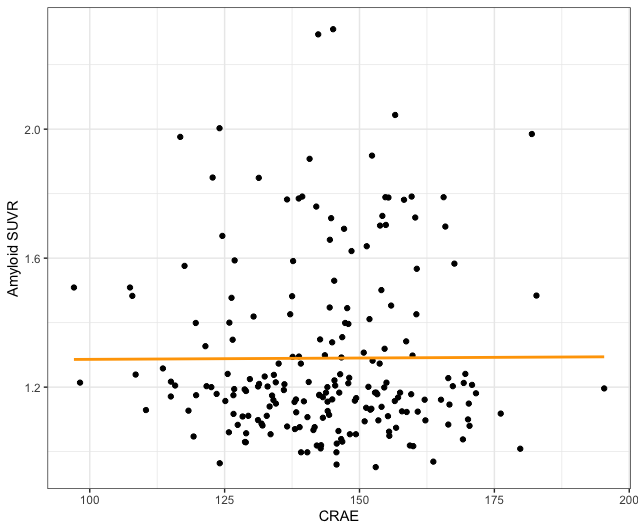


SUVR- standardized uptake value ratio, CRAE- central retinal arteriolar equivalent

**Supplementary Table 10. Associations, in separate models, between retinal signs at visit 5 and amyloid burden.** Model 1 was adjusted by the covariates age, sex, education, race and APOE-4 status. In Model 2 diabetes and hypertension were added as additional confounders at visit 5.

|  | Amyloid burden (continuous) | | | | | | | |
| --- | --- | --- | --- | --- | --- | --- | --- | --- |
|  | Model 1 | | | | Model 2 | | | |
|  | Beta  (95% CI) ^a^ | Beta  (95% CI) ^a^ | Beta  (95% CI) ^a^ | Beta  (95% CI) ^a^ | Beta  (95% CI) ^a^ | Beta  (95% CI) ^a^ | Beta  (95% CI) ^a^ | Beta  (95% CI) ^a^ |
| Age | **0.01**  **(0.00, 0.01)** | **0.01**  **(0.00, 0.01)** | **0.01**  **(0.00, 0.01)** | **0.01**  **(0.00, 0.01)** | **0.01**  **(0.00, 0.01)** | **0.01**  **(0.00, 0.01)** | **0.01**  **(0.00, 0.01)** | **0.01**  **(0.00, 0.01)** |
| Education |  |  |  |  |  |  |  |  |
| Less than high school | - | - | - | - | - | - | - | - |
| High school or comparable | -0.12  (-0.28, 0.01) | -0.10  (-0.26, 0.03) | -0.10  (-0.25, 0.04) | -0.10  (-0.25, 0.03) | -0.11  (-0.26, 0.02) | -0.09  (-0.24, 0.05) | -0.09  (-0.24, 0.05) | -0.09  (-0.24, 0.04) |
| At least some college | -0.10  (-0.26, 0.03) | -0.09  (-0.24, 0.05) | -0.09  (-0.23, 0.05) | -0.09  (-0.24, 0.04) | -0.10  (-0.26, 0.04) | -0.09  (-0.24, 0.06) | -0.09  (-0.23, 0.06) | -0.09  (-0.24, 0.05) |
| Sex |  |  |  |  |  |  |  |  |
| Female | - | - | - | - | - | - | - | - |
| Male | 0.03  (-0.03, 0.10) | 0.03  (-0.04, 0.09) | 0.03  (-0.03, 0.10) | -0.03  (-0.03, 0.10) | 0.03  (-0.03, 0.10) | 0.03  (-0.04, 0.09) | 0.03  (-0.03, 0.10) | 0.03  (-0.03, 0.10) |
| APOE-4 |  |  |  |  |  |  |  |  |
| Carrier | **0.19**  **(0.11, 0.27)** | **0.20**  **(0.12, 0.28)** | **0.20**  **(0.12, 0.28)** | **0.20**  **(0.12, 0.28)** | **0.19**  **(0.11, 0.27)** | **0.20**  **(0.12, 0.28)** | **0.20**  **(0.12, 0.28)** | **0.20**  **(0.12, 0.28)** |
| Race |  |  |  |  |  |  |  |  |
| Black | **0.10**  **(0.03, 0.18)** | **0.09**  **(0.02, 0.17)** | **0.09**  **(0.02, 0.16)** | **0.09**  **(0.02, 0.17)** | **0.10**  **(0.02, 0.18)** | **0.10**  **(0.02, 0.17)** | **0.09**  **(0.02, 0.17)** | **0.10**  **(0.02, 0.18)** |
| Hypertension |  |  |  |  |  |  |  |  |
| Present | - | - | - | - | -0.02  (-0.11, 0.05) | -0.02  (-0.10, 0.05) | -0.02  (-0.10, 0.05) | -0.02  (-0.10, 0.05) |
| Diabetes |  |  |  |  |  |  |  |  |
| Present | - | - | - | - | 0.05  (-0.02, 0.14) | 0.08  (0.01, 0.16) | 0.08  (0.01, 0.16) | 0.08  (0.01, 0.15) |
| Any retinopathy |  |  |  |  |  |  |  |  |
| Present | **0.18**  **(0.07, 0.36)** |  |  |  | **0.16**  **(0.02, 0.32)** |  |  |  |
| Arteriovenous nicking |  |  |  |  |  |  |  |  |
| Present |  | 0.01  (-0.10, 0.12) |  |  |  | 0.02  (-0.10, 0.12) |  |  |
| Focal arterial narrowing |  |  |  |  |  |  |  |  |
| Present |  |  | 0.06  (-0.09, 0.18) |  |  |  | 0.06  (-0.10, 0.17) |  |
| Generalized arteriolar narrowing |  |  |  |  |  |  |  |  |
| Present |  |  |  | -0.01  (-0.08, 0.06) |  |  |  | -0.01  (-0.06, 0.07) |

**Statistically significant associations p <0.05 marked in bold*

*^a^ 95% CI were computed using bootstrapping with 2000 bootstrap replicates*

|  | Amyloid (continuous) | |
| --- | --- | --- |
|  | Model 1 | Model 2 |
|  | Beta  (95% CI)^a^ | Beta  (95% CI)^a^ |
| Age | **0.01**  **(0.01, 0.01)** | **0.01**  **(0.01, 0.01)** |
| Education |  |  |
| Less than high school | - | - |
| High school or comparable | -0.12  (-0.28, 0.01) | -0.11  (-0.26, 0.02) |
| At least some college | -0.11  (-0.26, 0.03) | -0.10  (-0.26, 0.04) |
| Sex |  |  |
| Female | - | - |
| Male | 0.04  (-0.03, 0.11) | 0.03  (-0.03, 0.10) |
| APOE-4 |  |  |
| Carrier | **0.19**  **(0.11, 0.27)** | **0.19**  **(0.11, 0.27)** |
| Race |  |  |
| Black | **0.09**  **(0.02, 0.17)** | **0.10**  **(0.02, 0.18)** |
| Diabetes |  |  |
| Present | **-** | 0.05  (-0.03, 0.14) |
| Hypertension |  | -0.02  (-0.11, 0.05) |
| Present | **-** |  |
| Retinal scores |  |  |
| Retinal score = 1 | -0.02  (-0.08, 0.06) | -0.01  (-0.08, 0.06) |
| Retinal score= 2 | 0.02  (-0.14, 0.23) | 0.02  (-0.14, 0.23) |
| Retinal score= 3 | **0.19**  **(0.07, 0.34)** | **0.16**  **(0.04, 0.32)** |

**Supplementary Table 11. Association between retinal scores at visit 5 and increased amyloid burden at visit 5**. Model 1 was adjusted by age, sex, education, race, APOE-4. The association in model 2 was further adjusted by diabetes and hypertension.

*Statistically significant associations p <0.05 marked in bold

^a^ 95% CI were computed using bootstrapping with 2000 bootstrap replicates

**Supplementary Figure 2.** Boxplot showing the distribution of SUVR amyloid by retinal score. SUVR amyloid was significantly higher in those participants with a retinal score of 3.


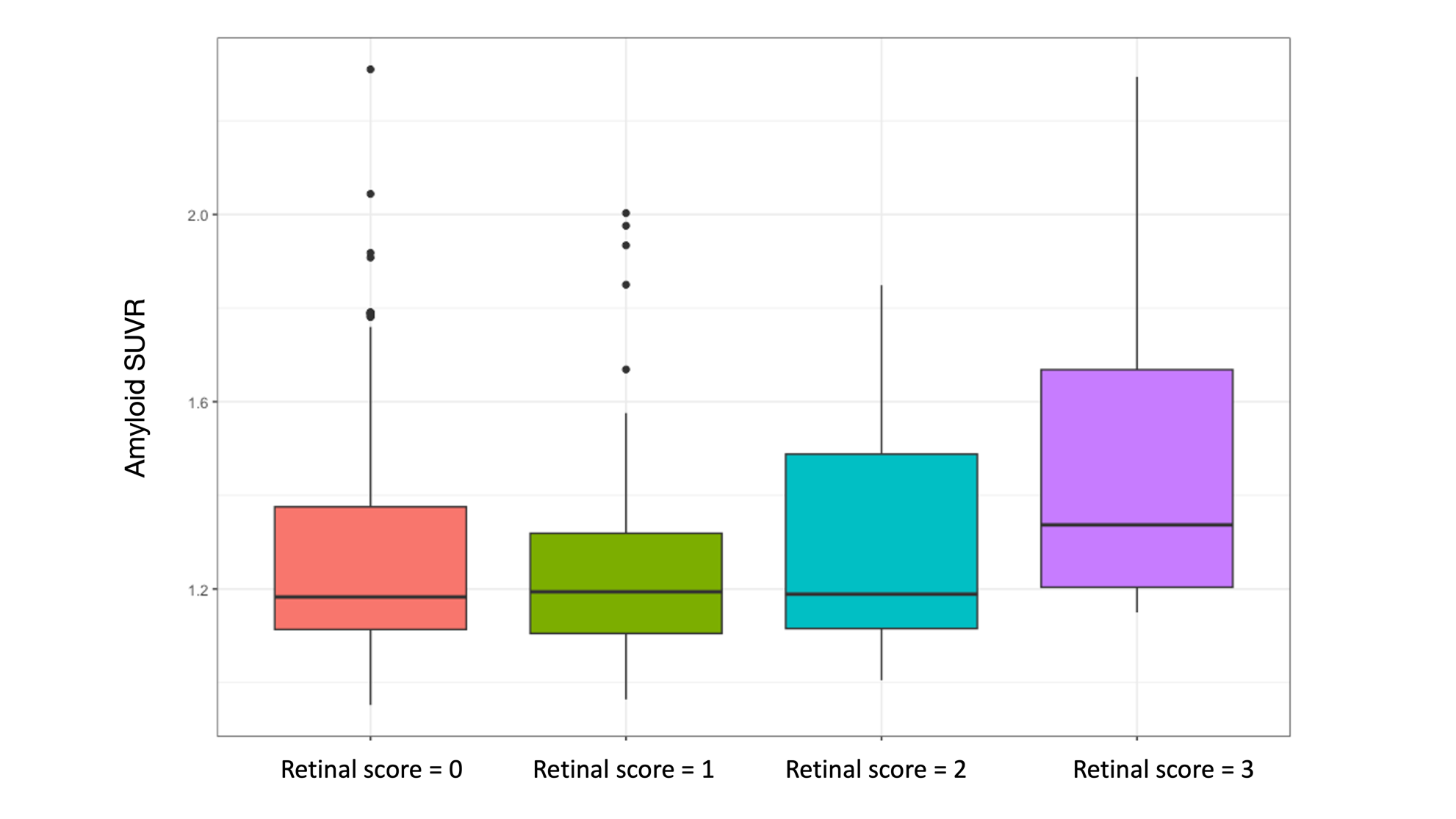


**Supplementary Table 12.** **Association between weighted retinal scores at visit 5 and increased amyloid burden at visit 5**. Model 1 was adjusted by age, sex, education, race, APOE-4. The association in model 2 was further adjusted by diabetes and hypertension.

|  | **Model 1** | **Model 2** |
| --- | --- | --- |
|  | Beta  (95% CI)^a^ | Beta  (95% CI)^a^ |
| Age | 0.01  (0.001, 0.012) | 0.01  (0.001, 0.013) |
| Education |  |  |
| Less than high school | — | — |
| High school or comparable | -0.12  (-0.27, 0.02) | -0.11  (-0.25, 0.03) |
| At least some college | -0.10  (-0.26, 0.04) | -0.10  (-0.25, 0.04) |
| Sex |  |  |
| Female | — | — |
| Male | 0.03  (-0.03, 0.10) | 0.03  (-0.03, 0.10) |
| APOE-4 |  |  |
| Carrier | 0.19  (0.11, 0.27) | 0.19  (0.12, 0.27) |
| Race |  |  |
| Black | 0.09  (0.02, 0.17) | 0.10  (0.03, 0.18) |
| Diabetes |  |  |
| Present | - | 0.05  (-0.02, 0.13) |
| Hypertension |  |  |
| Present | - | -0.02  (-0.10, 0.05) |
| Weighted retinal scores |  |  |
| Retinal score = 1 | -0.01  (-0.08, 0.06) | 0.00  (-0.08, 0.07) |
| Retinal score= 2 | 0.07  (-0.10, 0.24) | 0.07  (-0.09, 0.25) |
| Retinal score= 3 | **0.18**  **(0.07, 0.39)** | **0.16**  **(0.03, 0.34)** |

*Statistically significant associations p <0.05 marked in bold

^a^ 95% CI were computed using bootstrapping with 2000 bootstrap replicates
